# Food reward system: current perspectives and future research needs

**DOI:** 10.1093/nutrit/nuv002

**Published:** 2015-04-09

**Authors:** Miguel Alonso-Alonso, Stephen C. Woods, Marcia Pelchat, Patricia Sue Grigson, Eric Stice, Sadaf Farooqi, Chor San Khoo, Richard D. Mattes, Gary K. Beauchamp

**Affiliations:** *M. Alonso-Alonso* is with the Center for the Study of Nutrition Medicine, Beth Israel Deaconess Medical Center, Harvard Medical School, Boston, Massachusetts, USA. *S.C. Woods* is with the Department of Psychiatry and Behavioral Neuroscience, University of Cincinnati, Cincinnati, Ohio, USA. *M. Pelchat* and *G.K. Beauchamp* are with the Monell Chemical Senses Center, Philadelphia, Pennsylvania, USA. *P.S. Grigson* is with the Department of Neural and Behavioral Sciences, Penn State College of Medicine, Hershey, Pennsylvania, USA. *E. Stice* is with the Department of Psychology, University of Texas at Austin, Austin, Texas, USA. *S. Farooqi* is with the Wellcome Trust-MRC Institute of Metabolic Science, University of Cambridge, Cambridge, United Kingdom. *C.S. Khoo* is with the North American Branch of the International Life Sciences Institute, Washington, DC, USA. *R.D. Mattes* is with the Department of Nutrition Science, Purdue University, West Lafayette, Indiana, USA.

**Keywords:** addiction, craving, definitions, food reward system, palatable food, translational science

## Abstract

This article reviews current research and cross-disciplinary perspectives on the neuroscience of food reward in animals and humans, examines the scientific hypothesis of food addiction, discusses methodological and terminology challenges, and identifies knowledge gaps and future research needs. Topics addressed herein include the role of reward and hedonic aspects in the regulation of food intake, neuroanatomy and neurobiology of the reward system in animals and humans, responsivity of the brain reward system to palatable foods and drugs, translation of craving versus addiction, and cognitive control of food reward. The content is based on a workshop held in 2013 by the North American Branch of the International Life Sciences Institute.

## INTRODUCTION

**Figure 1:**
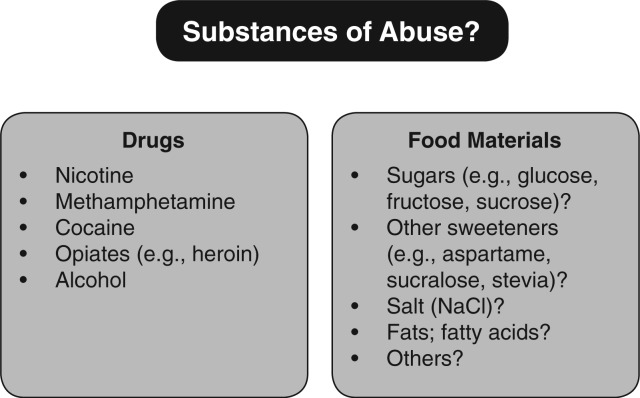
**Substances of abuse?** Science has yet to determine all of the mechanisms of action that may differentiate foods from drugs with regard to craving, dependence, tolerance, and abuse.

Growing knowledge on the role of the human food reward system in the regulation of food intake, along with the speculated link between the food reward system and addiction, has spurred increased interest and research within the scientific community. Many common food substances have been compared to drugs typically abused by humans, such as nicotine, alcohol, marijuana, methamphetamine, cocaine, and opioids (Figure 1). These drugs have often been associated with habitual use characterized by recurrent negative consequences (abuse) and physiological dependence (tolerance). More recent questions center on whether food substances (e.g., sugars, sweeteners, salt, and fats) can prompt similar addictive processes. The hedonic properties of food can stimulate feeding even when energy requirements have been met, contributing to weight gain and obesity.^1^ The latest national estimates of childhood and adult obesity in the United States show that, after 3 decades of growth, obesity rates have leveled off in the last decade.^2^ Yet the prevalence of obesity remains very high, putting Americans at risk for a wide range of health problems and adding to the nation’s healthcare costs.

Drugs and palatable foods share several properties. Both have powerful reinforcing effects that are mediated, in part, by abrupt dopamine increases in the brain reward system.^3^ This review focuses on these similarities and the potential impact of hedonic responses to foods on ingestive behavior, energy intake, and obesity. Topics addressed include the hedonic contribution to food intake regulation in humans, neuroanatomy and general principles of the brain reward system, brain reward responses to food as well as parallels between food and drugs, genetic contributions to overeating and obesity, cognitive control of food reward, translational applications, and challenges in defining “addiction” in the case of food. Although this work advances clarification of the concept of food addiction and its etiology, manifestations, and management, it is clear that critical questions about the specific pathways and parallel cue responses between drugs and food substances as well as their effects on intake behavior remain unanswered and require future research in humans.

## HEDONIC CONTRIBUTION TO REGULATION OF FOOD INTAKE IN HUMANS

Obesity prevalence and per capita food consumption in the United States have increased dramatically since the late 1970s,^4^ underscoring the need to more fully understand the neuronal substrates that underlie food intake. The regulation of food intake involves a close interrelationship between homeostatic and nonhomeostatic factors. The former are related to nutritional needs and monitor available energy within the blood and fat stores, whereas the latter are considered unrelated to nutritional or energy requirements, although both types of factors interact in key brain circuits. Maintaining a constant energy balance requires a very precise level of control: even a subtle but sustained mismatch between energy intake and energy expenditure can cause weight gain.^5^ A positive balance of as few as 11 calories a day over every day's energy need (which increases with weight), or approximately 4000 kcal per year,^6^^–8^ could result in a 1-pound gain over a year in an average-weight person. To sustain weight gain over years, a positive balance must be sustained that results in substantive increments in absolute intake (as observed in the general population, in which intake has risen by >200 kcal/d over the past 35 y); however, the balance only needs to be positive by a small amount on a daily basis.

**Figure 2:**
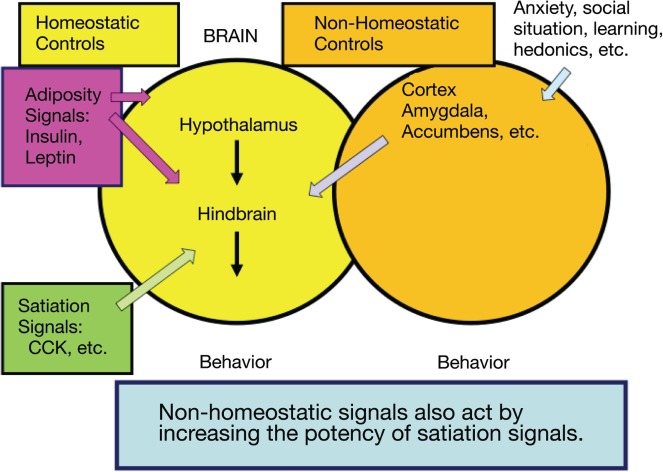
**Homeostatic and nonhomeostatic influences in regulation of food intake**. Food intake is determined by interplay between complex homeostatic and nonhomeostatic controls. *Abbreviation:* CCK, cholecystokinin.

Experimental studies in controlled environmental conditions (e.g., animals in laboratory settings) suggest that there are homeostatic factors that match energy intake with energy required to precisely control body weight over long periods of time.^9^ By contrast, population data from epidemiological studies indicate a robust tendency for weight gain in humans. In the past 30 years, adult obesity rates have more than doubled, from 15% in 1976 to 35.7% in 2009–2010. The average American adult is more than 24 pounds heavier today than in 1960,^10^ and 68.7% of US adults are either overweight or obese.^11^ This gain in average weight most likely reflects a change in the environment. It also suggests that, over time, nonhomeostatic contributors to food intake can be more influential than homeostatic ones (Figure 2).

Most nonhomeostatic mechanisms are related to the brain’s reward system. Understanding their role is a priority in this field of research. Until recently, most studies focused on the role of appetite regulation and homeostatic signals such as metabolic hormones and the availability of nutrients in the blood.^12^ However, interest in understanding how animals and humans eat in a nonregulated manner, or beyond metabolic needs, has become a priority in recent years.^12^ The sections that follow discuss the neurotransmitter dopamine, which is produced in the midbrain and stimulates the limbic areas such as the nucleus accumbens. Dopamine has emerged as a major nonhomeostatic influence over food intake.

Signaling mechanisms that initiate a meal are generally nonhomeostatic, whereas those that determine meal size are often homeostatic (i.e., the factors that influence when a meal will begin are qualitatively different from those that determine when a meal will end). Anticipated meals are preceded by a neurally controlled, coordinated secretion of hormones that prime the digestive system for the anticipated energy load^13^ and are modulated by perceived reward, learning, habits, convenience, opportunity, and social factors. By contrast, meal cessation (i.e., meal size and the feeling of fullness or satiation) is controlled in part by signals from the gastrointestinal tract (e.g., cholecystokinin, glucagon-like peptide-1, ghrelin, apolipoprotein A-IV, peptide YY) in proportion to ingested nutrients, and in part by nonhomeostatic signals.^9^ Some hormonal mediators (e.g., ghrelin and leptin) act through coordinated influences in brain regions involved in both homeostatic and nonhomeostatic regulation.

Homeostatic control over food intake is usually secondary to nonhomeostatic control, even for determining how much a person will eat in any given meal. These signals are probabilistic and are easily modified by nonhomeostatic factors. The ever-increasing availability of energy-dense and highly palatable foods over the last few decades demonstrates the influence that reward-related signals can exert. Essentially, reward-related signals can override homeostatic signals that would otherwise act to maintain a stable weight, thereby contributing to overeating.^13^

Drugs and foods share certain traits, but they also differ in qualitative and quantitative ways. Drugs of abuse, such as cocaine and amphetamine, directly influence brain dopamine circuits; other drugs influence similar brain circuits and also have direct, rapid access to the brain’s reward circuits. Foods influence the same circuits in two more indirect ways. The first is via neural input from the taste buds to dopamine-secreting neurons in the brain, and the second is through a later phase transmitted by hormones and other signals generated by the digestion and absorption of ingested food. The important point, however, is that the diverse influences over food intake and their oft-cited dichotomies (e.g., homeostatic vs nonhomeostatic or appetitive vs reward) are misleading because the controls are so completely interrelated at both the neural circuit level and in the specific neurotransmitters involved. Future studies need to directly assess these concepts by comparing the effect of drugs or foods in the same individual. Overall, better behavioral measures are needed to study the regulation of food intake in humans.

## THE BRAIN REWARD SYSTEM: NEUROANATOMY AND GENERAL PRINCIPLES

**Figure 3:**
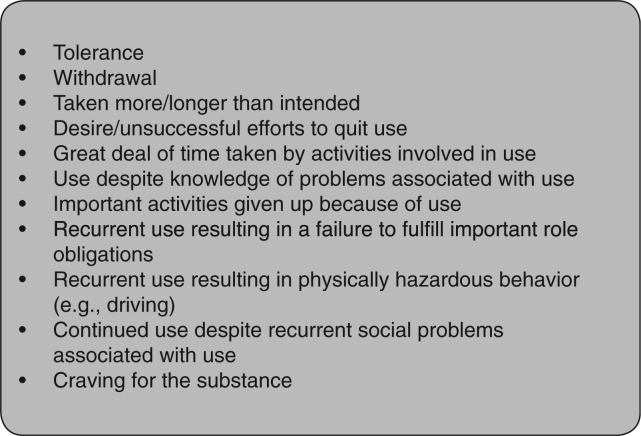
**DSM-5 criteria for substance use disorder**. Diagnosis is graded as mild (2–3 items), moderate (4–5 items), or severe (6 or more items).^14^

Almost anything in human experience can be rewarding, giving it the potential to become addictive, and this is evident across and within cultures. According to the 5th edition of the American Psychiatric Association’s *Diagnostic and Statistical Manual of Mental Disorders* (DSM-5),^14^ a diagnosis for addiction requires at least two of the following: withdrawal, tolerance, use of larger amounts of the substance over longer periods, spending a great deal of time obtaining and/or using the substance, repeated attempts to quit, activities given up, and continued use despite adverse consequences (Figure 3).^14^ Thus, like any other stimulus, food is suspect.

The neural system that mediates the experience of reward consists of a network of brain regions that studies show is growing in both number and complexity.^15^ The mesocorticolimbic pathway is a central component of this system. It arises from dopaminergic neurons located in the ventral tegmental area of the midbrain that send projections to target areas in the limbic forebrain, particularly the nucleus accumbens, as well as the prefrontal cortex.^16^ The prefrontal cortex, in turn, provides descending projections to the nucleus accumbens and the ventral tegmental area.^17^ This mesocorticolimbic circuit, then, is a key player in the final common pathway that processes reward signals and regulates motivated behavior in rats and, according to imaging data, in humans.^18^

In support of the central role proposed for the mesolimbic pathway, studies show elevated dopamine levels in the nucleus accumbens of rats following exposure to food,^19^ sweets,^20^ and sex.^21^ Self-administered drugs (e.g., cocaine, morphine, and ethanol) also lead to elevations in nucleus accumbens dopamine in rats.^22^ Dopamine levels are also higher with increasing concentrations of a sweet^23^ and a drug in rats.^22^ Finally, imaging studies in humans report activation of the striatum in response to food,^24^ drugs,^25^ money,^26^ and romantic love.^27^

Over time, humans and animals do not simply experience rewards: they anticipate them. As part of the learning process, dopamine levels in the nucleus accumbens and the activity of nucleus accumbens neurons are elevated in response to cues for food,^28^ sweets,^29^ sex,^21^ or drugs.^30^ Neural activity in the nucleus accumbens also increases in response to cues for larger vs smaller rewards.^29^ Like the rat brain, the human brain is also highly responsive to cues for food, drugs, or alcohol.^3^^,^^31^

In some cases, a cue may signal the immediate availability of a reward. In others, it may signal that a reward is imminent but that the subject will need to wait for access. Whereas cues that signal the immediate availability of a reward elicit increased levels of dopamine, those that signal a wait lead to reduced levels of nucleus accumbens dopamine in rats.^32^ Indeed, waiting for a drug is an adverse state in both rats and humans, and its onset is associated with devaluation of alternative rewards. Inattention to alternative rewards is a hallmark of addiction. Thus, rats avoid intake of an otherwise palatable saccharin cue while waiting for the opportunity to self-administer cocaine. The greater the avoidance of the taste cue, the more intense the drug taking.^33–35^ Likewise, humans waiting to smoke exhibit aversive affective behaviors and fail to elicit a normal striatal response to winning and losing money. Importantly, these outcomes were associated with greater cigarette seeking and taking in a two-choice test.^26^^,^^36^^,^^37^ Under these conditions, taking the drug (cocaine in the rodent studies and nicotine in the human studies) is the best correction for the conditioned aversive state, thereby reinforcing (i.e., “stamping-in”) continued drug-taking behavior via negative reinforcement.^38^

Individual responses vary greatly, and some humans and animals are more responsive than others. Therefore, it is possible to dramatically change one’s responsiveness to rewards, especially drugs, via experience. Drug and alcohol intake is greatly reduced after exposure to an enriched environment^39^ and access to a running wheel^40^ in rats, or after exposure to exercise in humans.^41^ By contrast, chronic sleep deprivation markedly augments the response to food stimuli in humans and the response to cocaine in rats.^42^^,^^43^ Likewise, in humans, there is a high comorbidity between substance abuse and eating disorders characterized by disinhibited eating.^44^ In rats, addiction-like behavior for cocaine is augmented (more than tripled) by a history of binging on fat,^45^ and responding for ethanol is augmented by a history of bingeing on sugar.^46^

In summary, dopamine not only tracks all natural rewards and drugs of abuse tested in rats and humans, it also tracks cues for these substances. Cue-induced anticipation of a highly palatable sweet^47^^,^^48^ or a drug of abuse^26^^,^^49^ leads to devaluation of lesser rewards. Indeed, cues for drugs elicit not only devaluation but also the onset of an aversive state when having to wait for access to the preferred reward. This state may involve conditioned craving and/or withdrawal. Recent data show that this conditioned aversive state can develop following a single drug exposure and can predict who will take a drug, when, and how much.^50^ Even so, as previously described, individual vulnerability can be reduced or augmented in rats and humans by a number of factors, including experience (e.g., the availability of an alternative reward, the opportunity to exercise, chronic sleep deprivation, or a history of binging on fat).

It is important to note that, across the range of human behavior, all manner of stimuli can become rewarding (e.g., sunbathing, shopping, gambling, piercing, tattooing, exercise, food, drink, sex, and drugs). Each of these stimuli, in turn, can support the development of addictive behavior, including seeking, taking, and/or engaging, sometimes at great cost. Some of these stimuli are potentially more addictive than others, and some individuals are more vulnerable. Food, like any other rewarding stimulus, thus has the potential to support the development of addictive behavior. Health, on the other hand, is promoted by moderation, the availability of alternate rewards, and balance across the realm of motivated behaviors.

## BRAIN REWARD RESPONSES TO FOOD AND PARALLELS WITH BRAIN REWARD RESPONSES TO DRUGS

Drugs of abuse and palatable foods show similarities in terms of how they engage reward circuitry in animals and humans. First, drugs activate reward-learning regions and dopamine signaling^51^; palatable food intake operates through the same pathway.^24^ Second, people escalate drug use due to tolerance, which is caused by plasticity changes in the dopaminergic system (downregulation of D2 receptors and upregulation of D1 receptors)^52^^,^^53^; intake of palatable food causes similar effects.^54^^,^^55^ Third, difficulties in quitting drug use are associated with hyper-responsivity in reward- and attention-related brain regions to drug cues^56^^,^^57^; obese subjects show a similar activation pattern when exposed to palatable food cues.^58^^,^^59^

Chronic drug use leads to neuroadaptation in reward circuits in a way that prompts escalation of intake. Animal experiments document that habitual intake of drugs of abuse results in a reduction of striatal D2 dopamine receptors and dopamine levels.^53^ Habitual intake also leads to the reduced sensitivity of reward regions to drug intake and electrical stimulation in experimental animals relative to control animals.^52^^,^^60^ These findings are consistent with cross-sectional data indicating that drug-dependent individuals show lower D2 receptor availability and reward region sensitivity, lower dopamine release from drugs, and reduced euphoria relative to findings in healthy controls.^61^^,^^62^ Likewise, animal experiments have documented that assignment to overfeeding vs nonoverfeeding conditions results in a reduction in D2 receptor availability, a reduction in dopamine availability and turnover, and reduced responsivity of reward regions to food intake, drug administration, and electrical stimulation.^54^^,^^63^

The above data are consistent with cross-sectional evidence that obese humans have fewer D2 receptors than lean humans and have a reduced reward region response to palatable food intake.^64^^,^^65^ In addition, longitudinal studies in humans suggest that this blunted brain reward response to food may be caused by overeating and weight gain.^66^ This conclusion is supported by experimental induction of obesity in animals such as rodents and pigs.^67^ Further evidence in humans comes from experimental studies in which participants were randomized to receive weight-stable or obesity-inducing palatable food on a daily basis. In the latter group, this resulted in decreased liking for the food, but increased wanting.^68^ Recent work suggests that the blunted responsivity in the striatum observed with functional magnetic resonance imaging (fMRI) in humans has high specificity. Subjects who report regular intake of ice cream show less reward region response to receipt of an ice-cream-based milkshake relative to adolescents who only eat ice cream rarely; consumption of other energy-dense foods, such as chocolate and candy, was unrelated to reward region response to ice cream receipt.^69^ This selectivity suggests parallels with the phenomenon of tolerance seen in drug addiction.

Another area of interest concerns the prediction of future weight gain. Studies in young humans at risk of weight gain suggest that elevated incentive salience, manifested as hyper-responsivity to food cues in brain areas related to reward valuation and attention, predicts future weight gain.^70–72^ This may be a maintenance factor that emerges after a period of overeating, rather than initial vulnerability. The mechanisms underlying the development of incentive sensitization appear to be related to initially elevated reward responses to palatable food and heightened associative learning capacity.^73^

**Figure 4:**
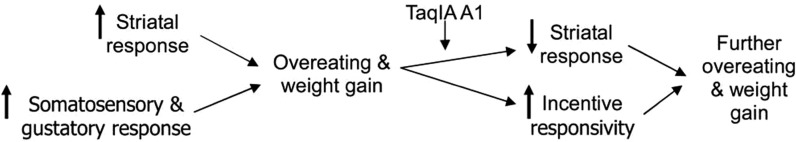
**Dynamic vulnerability model of obesity**. *TaqIA* refers to the single-nucleotide polymorphism of the *ANKK1* gene (rs1800497), which has 3 allelic variants: *A1/A1*, *A1/A2*, and *A2/A2.*

Taken together, the accumulated evidence is consistent with a dynamic vulnerability model in which individuals are at risk for obesity when initial hyper-reward responsivity from food intake leads to overeating, when striatal D2 receptor density and DA signaling become reduced in response to food intake, and when hyper-responsivity of regions that encode the incentive salience of food cues in a feed-forward fashion emerge^74^ (Figure 4).

In the future, brain imaging studies using repeated-measures designs might be useful for testing dynamic vulnerability hypotheses, such as whether heightened responsiveness to food cues predicts increased risk of future weight gain. The investigation of neuroscience-based prevention and treatment interventions (e.g., correcting a blunted striatal response to food) will be crucial, as will experimental confirmation of hypothesized relations.

The parallels between the neural effects of overeating and drug use are similar but not identical. Drugs of abuse lead to an artificial potentiation of dopamine signaling that does not occur in the case of food. Despite these and other differences, there are enough similarities to suggest that drugs and palatable food have the ability to engage the reward system in a way that promotes escalation of intake. However, it is not useful to determine whether certain foods are addictive; only a small number of people who try a pleasurable behavior become addicted. Instead, more productive routes are to focus on understanding the mechanisms by which drugs of abuse and palatable food engage the brain reward system toward escalated consumption, and to study individual differences that underlie the two contributing processes (blunted responses to the receipt of the food or drug, and hyper-responsivity of reward- and attention-related regions triggered by anticipatory cues). Finally, it might be more useful to consider the notion of food “abuse” rather than food “addiction” (i.e., implying dependence), because the evidence for dependence is somewhat mixed and inconclusive, but vast research clearly documents that obesity results in negative health and social consequences.

## GENETIC CONTRIBUTIONS TO OVEREATING AND OBESITY

Recent research indicates the critical role that human genetics plays in determining brain mechanisms of food reward. Studies in severe forms of obesity associated with extreme phenotypes of overeating provide a tractable approach to complex heterogeneous disorders such as obesity and diabetes. They can establish proof of principle of a single gene/pathway as well as insights into mechanisms that regulate body weight and associated phenotypes. This approach can advance drug discovery by validating old and new targets and setting the stage for stratified medicine. It can also deliver benefits for patients through advances in diagnosis, counseling, and interventions.

Twin, family, and adoption studies show that body weight is highly heritable. Common obesity is polygenic, with the genetic contribution to interindividual variation estimated at 40%–70%.^75^ Current molecular genetics has identified common DNA variants that affect body weight. Genome-wide association studies have investigated the genetic material of hundreds of thousands of individuals worldwide. However, all of the hereditary factors identified to date account for only about 5% of the variability of body mass index (BMI).^76^ Several rare highly penetrant genetic variants have been identified in severely obese patients, with associated changes in the brain reward system.

**Figure 5:**
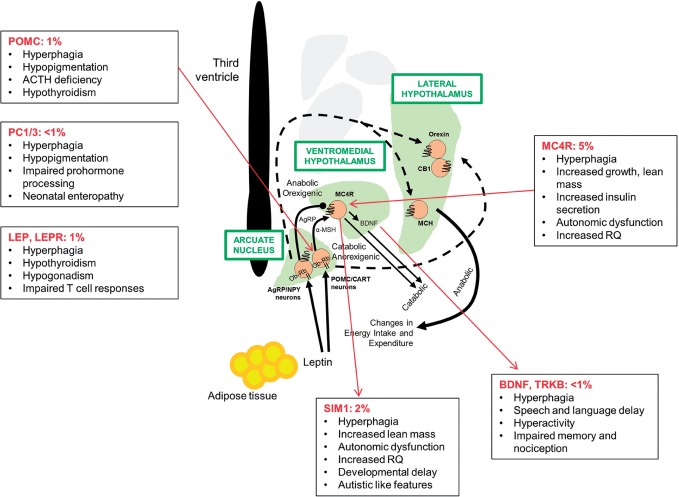
**Mutations in the leptin-melanocortin pathway in humans**. *Abbreviations*: ACTH, adrenocorticotropic hormone; AgRP, Agouti-related peptide; BDNF, brain-derived neurotrophic factor; CB1, cannabinoid type 1 receptor; incr., increased; LEP, leptin; LEPR, leptin receptor; MCH, melanin-concentrating hormone; *MC4R*, melanocortin 4 receptor gene; α-MSH, alpha-melanocyte-stimulating hormone; NPY, neuropeptide Y; Ob-Rb, leptin receptor, Ob-Rb isoform; PC1/3, prohormone convertase 1/3; POMC, pro-opiomelanocortin; RQ, respiratory quotient; SIM1, single-minded 1; TRKB, tyrosine kinase B.

Peptides and hormones, especially leptin, can act as modulators of energy balance. Leptin is a pivotal regulator of human energy balance through influences on brain regions involved in food reward. Leptin deficiency increases appetite and food intake. This hormone also modulates liking for food, which correlates with activation of the nucleus accumbens by dopamine. Known mutations in the leptin-melanocortin pathway in the hypothalamus lead to hyperphagia (Figure 5). Studies have evaluated phenotypes in patients with leptin deficiency using fMRI. In a seminal study, Farooqi et al.^77^ evaluated brain responses in 2 human patients with congenital leptin deficiency. Images of food before and after 67 days of leptin replacement therapy showed attenuation in neural activation of key striatal areas, suggesting that the therapy diminished the perception of food reward while enhancing the response to satiety signals generated during food consumption.^77^

Mutations in the melanocortin 4 receptor (*MC4R)* gene are the most common genetic cause of human obesity.^78^ Several treatment options (e.g., sibutramine, serotonin, and noradrenalin uptake inhibitors) have been investigated in human subjects with *MC4R* mutations. However, long-term body weight maintenance is rarely achieved.^78^ The use of fMRI data to compare striatal activation in 10 patients heterozygous for *MC4R* deficiency and 20 controls (10 obese and 10 lean) showed that *MC4R* deficiency was associated with altered striatal activation and food reward.^79^ This suggests that melanocortinergic tone may modulate the dopaminergic changes that occur with weight gain.

Additional genetic mutations, specifically those causing hyperphagia along with autonomic dysfunction, emotional lability, and autistic-type behavior, were recently linked to single-minded 1 – a basic helix-loop-helix transcription factor involved in the development and function of the paraventricular nucleus of the hypothalamus (Figure 5).^80^

Pharmacological manipulations of brain reward pathways in obesity use fMRI studies to examine correlates in the brain reward system associated with treatment outcomes following intake of sibutramine^81^ or a new µ-opioid receptor antagonist.^82^

There are likely more differences in the circuitry involved in drug reward vs food reward than currently proposed, which makes the case that obesity deserves to be studied in its own right. Attempted classification of foods as addictive is generally unhelpful. Rather, understanding the neural contribution to eating in different phenotypes is a critical step to making progress in the field. There is a need to develop tools to better define behavioral heterogeneity in a sensitive and objective manner as well as to understand the biology of the underlying behavior.

## COGNITIVE CONTROL OF FOOD REWARD: TRANSLATIONAL APPLICATIONS

In humans, behavioral drives for palatable food are moderated by cognition, specifically executive functions. These high-level mental functions support self-regulation of eating behavior and map to networks that include lateral and dorsomedial regions of the brain such as the dorsolateral prefrontal cortex, the dorsal anterior cingulate, and the parietal cortex. The environment in which we live challenges our limited physiological resources to suppress food intake. A central dilemma in daily living involves balancing one’s internal goals (i.e., knowledge, principles, or norms used to guide behavior, such as eating well to stay healthy or control weight) with the consequences of consuming food that is appetizing and immediately available. This conflict is particularly challenging with foods that are desired or craved; the interplay between cognition and reward is a fundamental component of the regulation of food intake in humans.

Recent studies with fMRI illustrate the ability to suppress the rewarding effects of food. These reports showed recruitment of brain regions related to executive functions/cognitive control when participants were asked to imagine delaying consumption of palatable foods shown in pictures or to think about the long-term benefits of not eating that specific food.^83^ Similar engagement of these brain regions is seen when men are asked to voluntarily suppress hunger.^84^ There is also evidence that food cravings interfere with competing cognitive demands, owing to an automatic direction of cognitive resources to craving-related cues,^85^ and thus attentional biases toward unhealthy food can predict an increase in BMI over time.^86^

Engagement of the lateral sectors of the prefrontal cortex may be a neural signature of compensatory mechanisms to overcome an individual’s tendency to overeat and gain weight. Observational studies have shown higher activation in these brain regions in successful weight-loss maintainers vs less-successful obese subjects.^87^^,^^88^ This finding shares some similarities with what is observed in the field of alcoholism, as unaffected first-degree relatives of alcoholics show strong prefrontal activity at rest, even at a higher level than that of healthy individuals.^89^ Due to limited longitudinal and experimental data, the specific directionality of the link between overeating/obesity and cognition is only partially known. Prospective studies report that individuals with reduced performance in tests that measure executive functions, particularly inhibitory control, show greater likelihood of future weight gain.^90^ However, added weight could also impair or interfere with these compensatory mechanisms, creating a vicious cycle. Growing cross-sectional evidence shows that obesity (BMI >30 kg/m^2^) is associated with impaired cognitive performance, including executive functions, attention, and memory.^91^ Even brain perfusion at rest is negatively correlated with BMI in regions related to executive functions, such as the cingulate cortex.^92^ This is also seen in animal models of experimental obesity.^67^ Weight loss is linked to small improvements in executive function and memory in obese (but not overweight) individuals.^93^ Accumulated evidence from neurocognitive tests and personality literature suggests that lateral prefrontal regions underpinning self-regulation, together with striatal regions implicated in food motivation, are critical neural systems related to individual differences in eating behavior and vulnerability to obesity.^94^

Many potential strategies could be used in the future to enhance the activity of brain regions related to cognitive control, including cognitive-behavioral therapy, cognitive training, exercise, noninvasive brain stimulation, neurofeedback, dietary modification, and medications. Although this field is still young, it is possible that certain foods or nutritional products could at least facilitate such brain changes. Neuroscience techniques can be used to screen potential compounds or interventions, providing information that is objective and sensitive.

Recent randomized placebo-controlled studies report increased activation of lateral prefrontal regions with 8-week intake of docosahexaenoic acid omega-3 supplements in children,^95^ 7-day intake of essence of chicken supplements in healthy elderly individuals,^96^ and a 24-hour high-nitrate diet (leafy green vegetables and beetroot juice) in elderly subjects.^97^ These results illustrate the potential modulatory role of foods and nutrients on brain regions that might facilitate control over food reward. Conversely, Edwards et al.^98^ report that eating a high-fat (74% kcal) diet for 7 days blunted cognitive function in sedentary men. Alternative strategies to enhance the contribution of cognitive control on food intake include the combination of cognitive training and noninvasive brain stimulation.^99^

**Figure 6:**
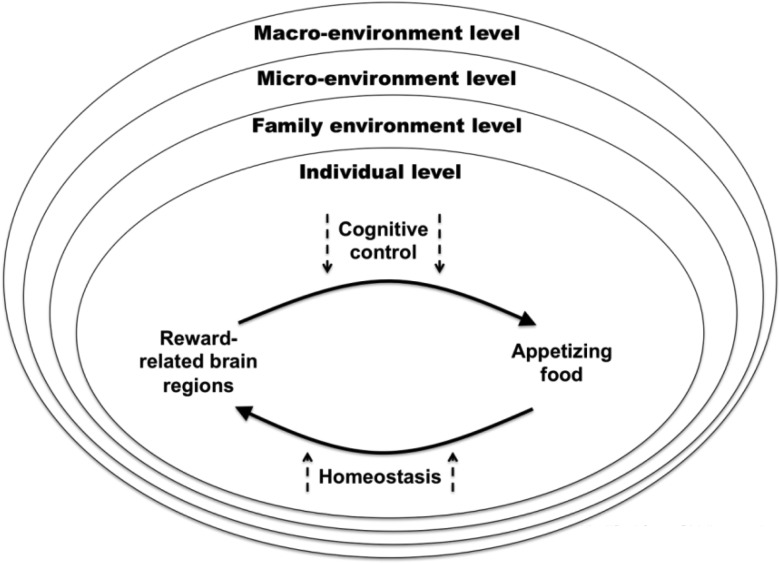
**Cognitive control of food reward and environmental influences**. Regulation of food intake, particularly the modulatory effect of cognitive control over food reward, occurs within the context of multiple levels of environmental influences. According to Gidding et al. (2009),^100^ there are 4 levels of influence: the individual level (level 1) is nested within the family environment (level 2) and is influenced by elements such as role modeling, feeding style, provision, and availability of foods, and so forth; the microenvironmental level (level 3) refers to the local environment or community and includes local schools, playgrounds, walking areas, and shopping markets that enable or impede healthful eating behaviors; and the macroenvironmental level (level 4) refers to broader regional, state, national, and international economic and industry policies and laws, which can affect individual choices. Gidding et al. (2009)^100^ state that this model “recognizes the importance of both the nesting of levels within one another and reciprocal influences among levels.”

Interactions between the brain systems associated with cognition, reward, and homeostasis do not occur in isolation; rather, they are embedded in the environment and the situational factors that result from it (Figure 6).^100^ A need exists for more studies performed in ecologically valid settings as well as research that can integrate aspects close to the real-life individual–food interaction. For instance, little is known about how cultural values shape the food reward system, which likely happens via brain substrates of cognition. Culturally determined attitudes and views on food may influence the processing and expression of food reward.

In general, the field warrants methodological innovations to bring scientific advances from the laboratory to the clinic. These include emerging neurotechnologies such as portable, noninvasive tools and computerized assessments to examine key neurocognitive components of eating behavior. These methodologies can help build a base of knowledge on the impact of nutrients, food products, and diets on the brain relative to healthy eating and weight control.

## CHALLENGES IN DEFINING “ADDICTION” IN THE CASE OF FOOD

Numerous sources of common confusion are related to the term “addiction” and center on the following four words: liking, reward, wanting, and craving. Liking is defined as the hedonic response to or the pleasantness of a stimulus. Reward is often assumed to be synonymous with pleasure but is defined by behaviorists as that which enhances the act that preceded it. Thus, reinforcers can operate without conscious awareness or pleasure (e.g., energy conditioning in postingestive learning). Wanting is equivalent to desire. In its transition to being something desired, an object is said to have acquired incentive salience, which results from the pairing of reward with objects or cues. A craving is a very strong desire.

Food cravings (i.e., intense desires to eat particular foods) are extremely common^101^ and are not necessarily pathological. A food does not have to be delicious to be craved. Food cravings are correlated with high BMI as well as with behaviors that might lead to weight gain, including increased snacking, poor compliance with dietary restrictions, and binge eating/bulimia.^102^^,^^103^ By contrast, many believe that cravings reflect the “wisdom of the body” (i.e., a nutritional need). However, monotony or restriction in the absence of nutritional deficit can also bring on craving. In a study of young adults by Pelchat and Shaefer,^104^ subjects reported significantly more cravings during the monotony manipulation than during the baseline period.

Regarding the nature of food cravings, the type of food varies with culture. It is not known whether there are key food characteristics (e.g., palatability, energy, fat, or sugar content) that lead to craving, or whether it is the way in which the food is consumed (e.g., if it is perceived as forbidden, or if it is consumed in an intermittent, restricted manner). The role of restricted access in humans has just started to be experimentally assessed. For instance, this mechanism was proposed to explain the rise in sushi craving among Japanese women.^105^ Solving these questions is particularly important and could have implications for policy (e.g., whether sugary drinks or diets should be outlawed).

A seminal study used fMRI to examine brain activation during the induction of food cravings. Pelchat et al.^106^ found that changes occurred in the hippocampus, the insula, and the caudate – 3 sites involved in drug craving. However, activation in the same brain reward substrates is quite normal and can be observed for innocuous pleasurable stimuli, such as music.^107^ Such a pattern of brain activation does not imply addiction. Activation in brain reward pathways in response to food is a sensitive parameter with low specificity, because many sources of pleasure and motivated behaviors lead to activation of this system. Neuroimaging is useful for understanding mechanisms; however, it is not a valid methodology to diagnose addiction on its own.

The American Psychiatric Association has not recognized food addiction as either an eating disorder or a substance abuse disorder. However, the DSM criteria are being used as a food-addiction scale.^108^ To accept this measure, it is necessary to establish whether the diagnosis corresponds to a disordered response to all foods or to one particular type of food. It is also uncertain what the concepts of tolerance and withdrawal may mean for the case of food. Thresholds for dysfunction are also unclear and are undefined for food and for drugs. Ultimately, food addiction would be a diagnosis based on negative consequences of maladaptive behaviors, but food addiction itself does not cause anything.

## CONCLUSION

This review reveals several key findings. First, the regulation of food intake is complex and involves multiple levels of control through environmental cues and cognitive, sensory, metabolic, endocrine, and neural pathways. The rewarding properties of food can override basic satiation signals generated in homeostatic centers. Second, food and drugs engage overlapping brain reward pathways, and both elicit the release of dopamine. However, there are fundamental differences, both qualitative and quantitative. Commonly abused drugs artificially prolong dopamine signaling, whereas intake of palatable food does not. Third, addiction is determined by the subjective experience of an individual. A certain amount of dopamine release and activation of the brain reward system are not necessary or sufficient conditions for addiction. Finally, individual experiences and genetic variation underlie differences in how the brain responds to rewarding properties of foods. In real life, these brain responses are moderated by additional factors (e.g., reward alternatives, cognition, and environmental influences).

Listed below are several identified research needs that can be best addressed by collaborative approaches.

*Broadening the scope*. The scope of research in the field of food reward should be broadened toward evaluation of eating-behavior phenotypes and their brain/neurocognitive underpinnings and examination of the specificity of the food-addiction phenotype and its overall relevance/implications.
*Addiction mechanisms for food vs drugs.* Available information should be complemented with an expansion of research on differences between addiction and addiction-like mechanisms for foods and drugs. There are likely more differences in the circuitry involved in drugs vs food than what is currently known.
*Food reward vs intrinsic individual vulnerability.* The contribution of rewarding properties of food needs to be disentangled from intrinsic individual vulnerability factors, with interactions and dynamics between the 2 components determined. There is a need to identify foods or food characteristics that may be specific targets for rewarding and addictive behavior. Alternatively, can any food or, more likely, food ingredient be “addictive”? What are the contexts and experiences?
*Human eating behavior.* New methodologies and tools to better define and understand the heterogeneity of human eating behavior and the underlying biology, including the food-addiction phenotype, need to be developed. These methods should be reproducible and valid, providing sensitive and objective information. Specifically, it is necessary to identify and develop new markers that can differentiate the transitions from impulsive to compulsive to addictive behavior in the case of eating.
*Clarification of terminology and metrics.* Better agreement and harmonization of semantics, definitions, and metrics for describing variability in human eating behavior is needed. In particular, there is a need to clarify how the addiction concept and definition as indicated in DSM-5 (Figure 3)^14^ can be, or even should be, applied to foods. This is necessary to avoid mischaracterization of foods and/or other substances in the absence of agreement on validated metrics. It is necessary to establish clarity on whether the DSM-5 definition corresponds to a disordered response to all foods or to one particular type of food or ingredient. It is also uncertain what the concepts of tolerance and withdrawal may mean in the case of food. Thresholds for dysfunction are also unclear and undefined, as is the link with health consequences (e.g., obesity).
*Etiology, causality, and maintenance of overeating.* More research to inform causality of the etiologic processes that lead to overeating and the maintenance processes that sustain it in humans should be conducted. Further study is needed to elucidate the precise time course of dopamine responses and brain reward system activation. Experimental research, such as randomized controlled trials, can help determine whether food addiction and/or obesity are driving a change in reward value or vice versa.
*Evolution of food reward system.* Greater understanding of the evolutionary aspects of food reward in this context is needed. Did the human reward system evolve to anticipate and respond to foods, and thus to preserve survival, or has it been shaped/reshaped by the food environment, and if so, to what extent?

Finally, there is an overall need for innovative methods in the field to better evaluate the neurocognitive components of human eating behavior. The development of new methods in this area can enhance discovery and ultimately help build a base of knowledge on the impact of nutrients, food products, and diets on the brain. It can also provide the basis for new ways to stimulate inhibitory mechanisms as well to suppress activation mechanisms, with potential implications for the fields of food and nutrition, medicine, and public health.
